# Fluoride Chemistry in Tin Halide Perovskites

**DOI:** 10.1002/anie.202107599

**Published:** 2021-07-24

**Authors:** Jorge Pascual, Marion Flatken, Roberto Félix, Guixiang Li, Silver‐Hamill Turren‐Cruz, Mahmoud H. Aldamasy, Claudia Hartmann, Meng Li, Diego Di Girolamo, Giuseppe Nasti, Elif Hüsam, Regan G. Wilks, André Dallmann, Marcus Bär, Armin Hoell, Antonio Abate

**Affiliations:** ^1^ Helmholtz-Zentrum Berlin für Materialien und Energie GmbH Hahn-Meitner-Platz 1 14109 Berlin Germany; ^2^ Institute of Advanced Materials (INAM) Jaume I University Castelló de la Plana Spain; ^3^ Egyptian Petroleum Research Institute Cairo Egypt; ^4^ Department of Chemical, Materials and Production Engineering University of Naples Federico II 80125 Naples Italy; ^5^ Institut für Chemie Humboldt Universität zu Berlin Brook-Taylor-Str. 2 12489 Berlin Germany; ^6^ Department of Chemistry and Pharmacy Friedrich-Alexander Universität Erlangen-Nürnberg (FAU) 91058 Erlangen Germany; ^7^ Department for X-ray Spectroscopy at Interfaces of Thin Films Helmholtz-Institute Erlangen-Nürnberg for Renewable Energy (HIERN) 12489 Berlin Germany

**Keywords:** lead-free systems, perovskite solar cells, tin fluoride, tin halide perovskites, tin oxidation

## Abstract

Tin is the frontrunner for substituting toxic lead in perovskite solar cells. However, tin suffers the detrimental oxidation of Sn^II^ to Sn^IV^. Most of reported strategies employ SnF_2_ in the perovskite precursor solution to prevent Sn^IV^ formation. Nevertheless, the working mechanism of this additive remains debated. To further elucidate it, we investigate the fluoride chemistry in tin halide perovskites by complementary analytical tools. NMR analysis of the precursor solution discloses a strong preferential affinity of fluoride anions for Sn^IV^ over Sn^II^, selectively complexing it as SnF_4_. Hard X‐ray photoelectron spectroscopy on films shows the lower tendency of SnF_4_ than SnI_4_ to get included in the perovskite structure, hence preventing the inclusion of Sn^IV^ in the film. Finally, small‐angle X‐ray scattering reveals the strong influence of fluoride on the colloidal chemistry of precursor dispersions, directly affecting perovskite crystallization.

## Graphical abstract

Fluoride chemistry in tin halide perovskites improves the crystallization process. Fluoride anions selectively coordinate and remove Sn^IV^ and affect the colloidal properties in solution. This study describes the working mechanism of SnF_2_ and highlights the importance of solution chemistry for controlling crystallization and Sn^II^ oxidation in tin halide perovskites.

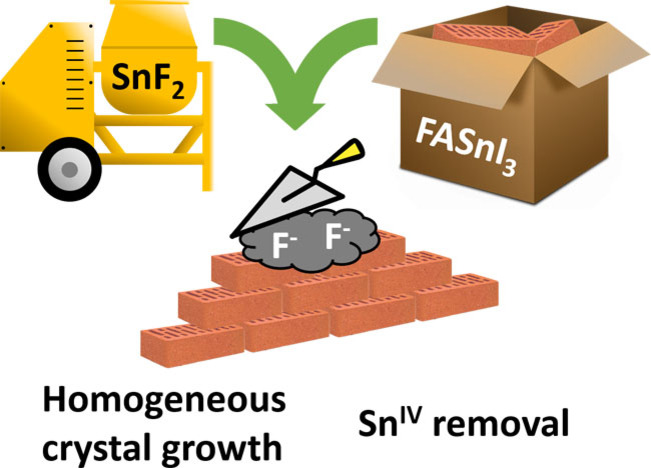

## Introduction

Metal halide perovskite materials have shown enormous potential for the processing of efficient and stable photovoltaics.^[1]^ However, the dominant type of perovskite solar cells (PSCs) are based on lead, a metal whose toxicity and environmental hazard can hinder its commercial application in numerous fields.^[2]^ The lead threat pushed the scientific community to develop lead‐free perovskite materials to maintain excellent photovoltaic performance while avoiding environmental risks. In this sense, tin halide perovskites are the best candidate to replace the dominant lead‐based counterparts.[^3^, ^4^] Nevertheless, these materials face some difficulties related to their inherent physicochemical characteristics.^[5]^ The most important one is the ease with which Sn^II^ oxidizes into Sn^IV^ species, leading to the substantial decline in the performance through the undesirable formation of electron traps and p‐doping of the material.^[6]^ Previous studies have reported many origins of this oxidation, such as the solvent,[^7^, ^8^] the processing conditions^[9]^ or even spontaneously through disproportionation in tin‐poor environments.^[10]^ Stopping this oxidation is one of the requirements to achieve efficient and stable tin halide PSCs. For this reason, several trials have been made tackling the oxidation of Sn^II^. These include the use of new solvent systems to avoid the oxidation by dimethyl sulfoxide (DMSO),^[11]^ employing reducing agents to eliminate the content of Sn^IV^, such as metallic Sn powder^[12]^ or hypophosphorous acid^[13]^ or introducing additives for alleviating the formation of Sn^IV^, like the ever‐present SnF_2_.[^6^, ^14^]

**Figure 1:**
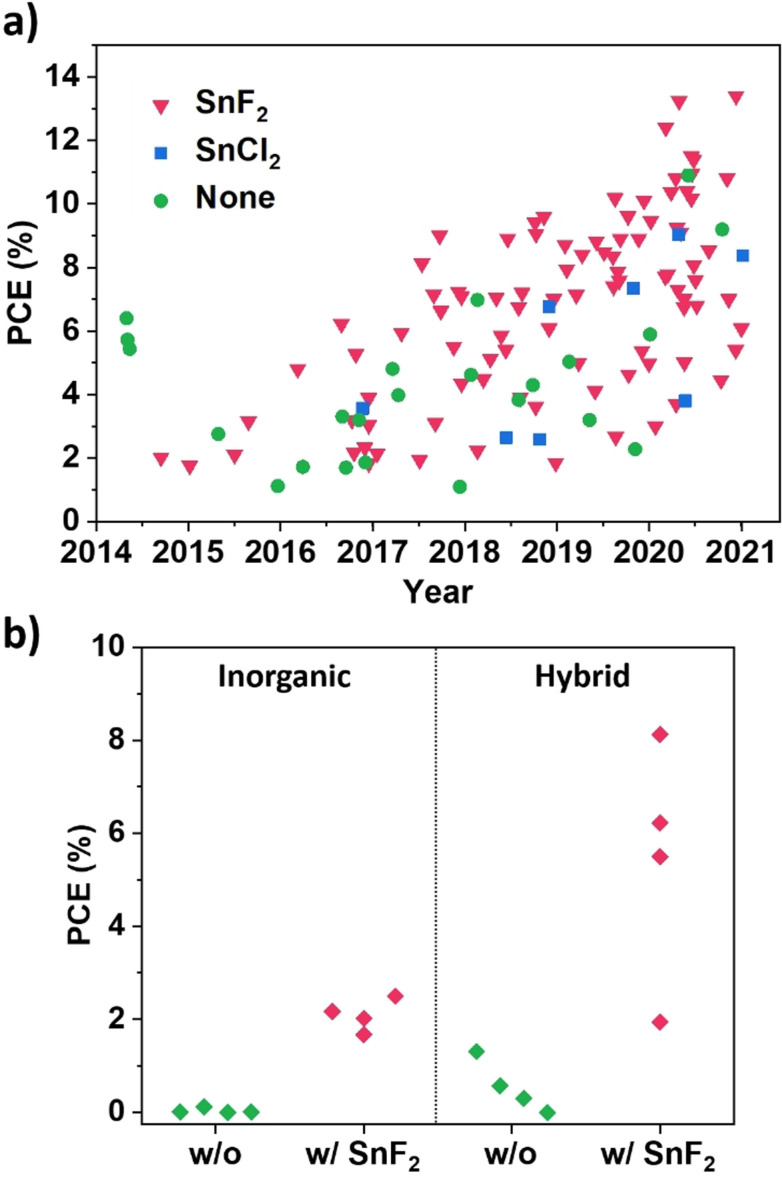
a) Highest PCE reported in tin halide perovskites literature containing solar cell data using SnF_2_, SnCl_2_, or none of them, ordered by date until Jan. 2021. b) Comparison of solar cell performance improvement in inorganic and hybrid tin halide perovskites before and after the addition of SnF_2_, extracted from the studies in literature in which the comparison is made.[^15^, ^21^, ^22^, ^23^, ^24^, ^25^, ^26^, ^27^]

SnF_2_ has achieved remarkable success as an additive in the tin halide perovskite field. Since its first use in PSCs by Kumar and co‐workers,^[15]^ it has been proven over time as an imperative to achieve good results (Figure 1 a). There is barely any good cell performance report without SnF_2_; exceptional cases use SnCl_2_,[^16^, ^17^] which may behave similarly to SnF_2_, or 2D materials, which are another popular strategy for processing tin‐based perovskites.[^18^, ^19^, ^20^] The appeal of SnF_2_ in the community is such that the number of studies not using it (nor SnCl_2_) quickly stagnated over the years, being in 2020 below 10 % of the total publications on tin halide perovskites in that year (Figure S1). Its success lies mainly in the impossibility to obtain photovoltaic behaviour in the solar cells fabricated without it. In Figure 1 b, we collected data from studies in which solar cells were made with and without SnF_2_.[^15^, ^21^, ^22^, ^23^, ^24^, ^25^, ^26^, ^27^] The improvement in efficiency for both inorganic and hybrid tin halide perovskites is enormous, with negligible efficiency for the SnF_2_‐free cases. One of the most reported improvements is the better substrate coverage and film morphology obtained with SnF_2_,[^22^, ^25^, ^28^] which implies that SnF_2_ affects the film crystallization, a factor that remains unexplored. Nevertheless, its addition needs to be controlled, as a too‐high content of SnF_2_ is reported to induce phase separation.[^22^, ^25^, ^28^] The other most explored effect is the ability of SnF_2_ to reduce the formation of Sn^IV^ and its related defects, with the reported benefits usually being reduced recombination^[24]^ and a blue shift of the absorption onset.[^24^, ^29^, ^30^] Besides, Savill and co‐workers found out that even low amounts of SnF_2_ are sufficient to positively impact mitigating Sn^IV^ formation in tin/lead perovskites.^[29]^ This effect on oxidation suppression could originate from introducing a Sn‐rich environment, reducing Sn vacancies.^[10]^ This interpretation has been proposed already in the first use of this additive by Kumar et al.^[15]^ However, the question of what fluoride is doing and why we do not provide the Sn‐rich environment simply with a higher SnI_2_ ratio in respect to FAI remains unanswered. Related to this, other Sn^II^ species that could provide the same beneficial effect were already discussed by Yokoyama et al.^[31]^ Using SnI_2_ excess also led to good results in one of the first studies on inorganic tin halide perovskites.^[32]^

Overall, reports in literature consistently lead to the same results: SnF_2_ has a critical positive influence on the formation of high‐quality ASnX_3_ (where A=methylammonium (MA^+^), formamidinium (FA^+^) and Cs^+^, and X=Cl^−^, Br^−^ and I^−^) films and holds a particular role in the stability of these materials against their oxidation to Sn^IV^. These two possibly related aspects are the key to SnF_2_ being the predominant, most robust additive in the tin‐based perovskite field. While there has been extensive exploration of the impact and functioning of SnF_2_ in these thin films, the chemical mechanism and influence in the processing are entirely unknown. Introducing an exact Scheme of its working procedure remains a must in the field. This move would open the door to optimized application and help identify new additives in the future.

In this work, we explain the origin of the beneficial effects of SnF_2_ in the processing and stability to oxidation of tin halide perovskites by studying the chemistry of fluoride in these solutions. Using a combination of complementary solution and film characterization techniques, we propose that the role of SnF_2_ is not limited to the resulting thin film but also affects the precursor solution properties critically and hence their processing. The study of the solution chemistry of fluoride in formamidinium (FA)‐based FASnI_3_ precursor solutions by ^119^Sn‐ and ^1^H‐NMR revealed a strongly predominant affinity of the fluoride anion for Sn^IV^ over Sn^II^. With the help of hard X‐ray photoelectron spectroscopy (HAXPES) analysis, we show how SnF_2_ increases the Sn^II^ content in perovskite samples, an indication that Sn^IV^ is partially prevented from being incorporated in the perovskite film. Meanwhile, small‐angle X‐ray scattering (SAXS) enables an understanding of how fluoride anion modifies perovskite subunits’ interaction in solution, generating improved homogeneous crystal growth conditions. Furthermore, experiments with other fluoride species and SnCl_2_ prove that these effects are not exclusive to SnF_2_. Thus, the chemistry of a hard Lewis base like fluoride, combined with the Sn‐rich environment, make SnF_2_ a highly suitable additive for processing tin halide perovskites.

## Results and Discussion

Previous works concluded that SnF_2_ could reduce the Sn^IV^ content in solutions and films.[^14^, ^30^] However, the redox activity cannot explain the multiple effects of SnF_2_ in ASnX_3_ perovskites entirely. Therefore, we postulated that SnF_2_ must be involved in a different type of chemical reaction. In the early stages of tin halide perovskites development, it was thought that a yellow color for the solution implied the elimination of Sn^IV^ through its reduction by SnF_2_.^[28]^ Using NMR, we uncover that Sn^IV^ and SnF_2_ do not undergo a redox reaction, but a simple ligand exchange reaction, producing colorless SnF_4_ in solution. In this regard, we prepared FASnI_3_ precursors solutions with and without SnF_2_ and Sn^IV^ to investigate their signature chemistry. ^119^Sn‐NMR is sensitive to Sn nuclei in different electronic environments, allowing to identify of the Sn species existing in the solution, including other oxidation states of the same nucleus.

**Figure 2:**
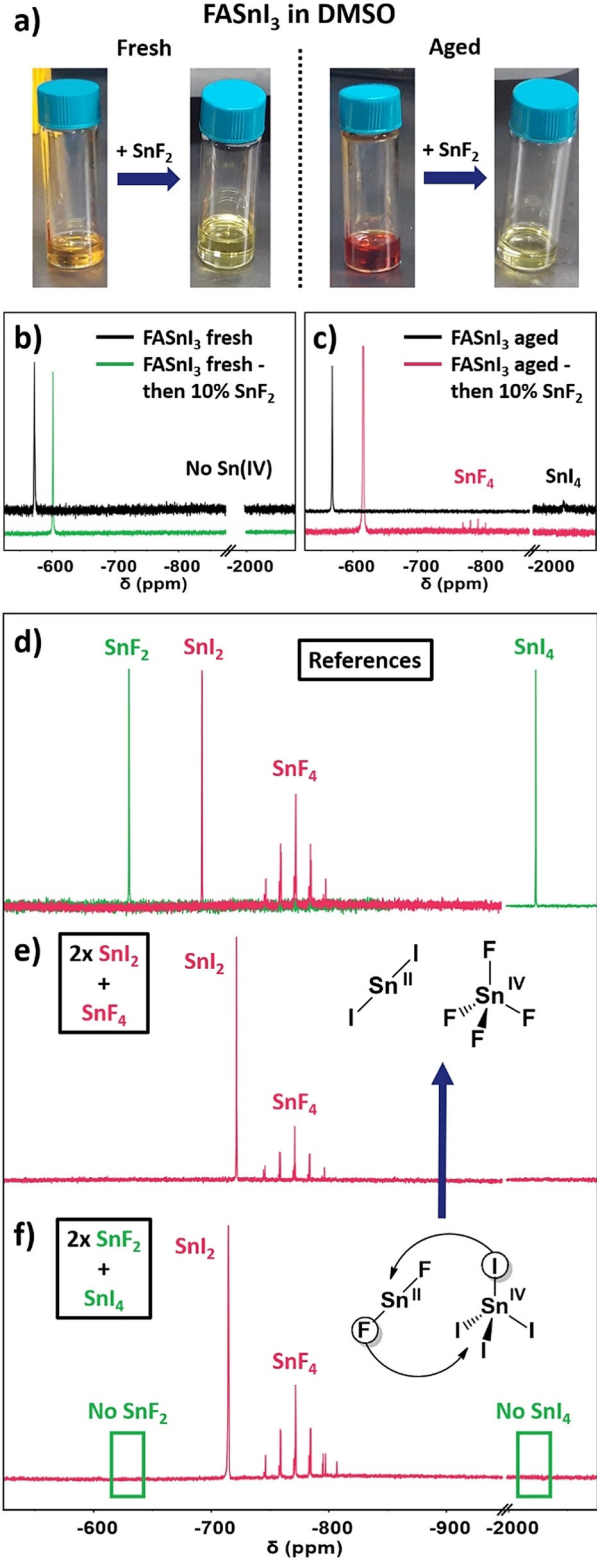
a) Pictures of fresh and aged FASnI_3_ solutions in DMSO before and after the addition of SnF_2_ in 10 mol %; the respective ^119^Sn‐NMR spectra of b) fresh and c) aged solutions; d) ^119^Sn‐NMR spectra of SnF_2_, SnI_2_, SnF_4_ and SnI_4_ in DMSO. Further information about solution preparation in Figure S3. ^119^Sn‐NMR spectra and solution pictures of the mixing of e) 2× SnI_2_ and SnF_4_ and f) 2× SnF_2_ and SnI_4_. The signal magnification was adapted accordingly for illustrative purposes.

Figure 2 a depicts the change in color from orange to the pale yellow of a 1 M FASnI_3_ solution in DMSO after the addition of SnF_2_. Following the same method, we added SnF_2_ to a Sn^IV^‐containing solution after being aged by heating at 100 °C for 3 h. Saidaminov et al. and we recently described that this thermal treatment promotes Sn^II^ oxidation in DMSO solutions.[^7^, ^8^] The color of the aged solution goes from an intense red to a pale yellow to that of the fresh sample. Unlike the fresh solution, we know that certain content of Sn^IV^ is present for the aged one. Still, the color after SnF_2_ for the aged solution is the same as for the fresh solution, suggesting that the characteristics and species in the solution are changing, no matter the Sn^IV^ content.

^119^Sn‐NMR of the fresh solution indicates neither elimination nor formation of Sn^IV^, even though its color changes to pale yellow (Figure 2 b). However, we observe the Sn^II^ shielding resulting in a chemical shift change from −574 ppm to −604 ppm. Regarding the Sn^II^ species (SnI_2_ and SnF_2_), they cannot be differentiated in solution, as they show up in a single signal belonging to the average electronic environment of Sn^II^ in solution. In this sense, the addition of increasing amounts of SnF_2_ shifts the Sn^II^ peak to lower chemical shift values in a fairly linear manner, as we show in Figure S2. In contrast to the fresh solution, the aged FASnI_3_ solution showed the expected SnI_4_ signal at −2025 ppm. After the addition of SnF_2_, this peak disappeared, and a new quintuplet rose at −770 ppm (Figure 2 c). To identify the newly formed species, we measured solutions in DMSO of SnI_2_, SnI_4_, SnF_2_ and SnF_4_ by ^119^Sn‐NMR (Figures 2 d), indicating that the species corresponds to SnF_4_. This result, therefore, implies that SnF_2_ cannot reduce Sn^IV^ from an oxidized sample. Instead, it coordinates Sn^IV^ via a ligand exchange reaction between the fluorides from Sn^II^F_2_ and the iodides from oxidized Sn^IV^I_4_ [Eq. 1]:

(1) $$\mathrm{Sn}{}^{\mathrm{IV}}I{}_{4}+2\;\mathrm{Sn}{}^{\mathrm{II}}F{}_{2}\to \mathrm{Sn}{}^{\mathrm{IV}}F{}_{4}+2\;\mathrm{Sn}{}^{\mathrm{II}}I{}_{2}$$

As shown in Figure 2 e, a colorless solution comprising SnI_2_ and SnF_4_ presented both mixed species’ signals in NMR. However, mixing SnF_2_ and SnI_4_, the resulting NMR species observed were SnI_2_ and SnF_4_. Consequently, the complexation selectivity of fluoride ions towards Sn^IV^ is absolute (Figure 2 f). This can be easily explained by the “hard and soft (Lewis) acids and bases” (i.e. HSAB theory) nature of the different solution species. Fluoride is a small, non‐polarizable, very electronegative anion that shows a stronger affinity for a cation of a similar nature, that is, Sn^IV^, which is smaller and more electronegative than its reduced analogue Sn^II^. This hard Lewis base character of fluoride anions was already applied in previous works on lead halide perovskites, owing to its ability to passivate vacancies due to their strong bonds with Pb^II^.^[33]^ In the present case, we find that fluoride's role is connected to Sn^IV^ complexation. Simultaneously, the passivation of undercoordinated Sn^II^ in the thin film should not be excluded.

The only appearance so far of this species can be found in Nakamura and co‐workers’ work. The authors use an SnF_2_‐selective reducing agent to effectively generate Sn^0^ nanoparticles to scavenge Sn^IV^ from the solution.^[34]^ Even though there is no particular discussion on the formation of SnF_4_ and it does not affect their mechanism, the ^119^Sn‐NMR spectra provided in their work show the signal, also a multiplet, corresponding to SnF_4_ at approximately −750 ppm when adding SnF_2_ to a Sn^IV^‐containing solution. This different multiplicity that the SnF_4_ signal presents in ^119^Sn‐NMR compared to the rest of the Sn species can be explained by coupling between the Sn and halide nuclei. SnF_4_ has four chemically equivalent ^19^F‐spins (each with spin 1/2) to couple with, which results in a perfect quintuplet as observed. In this sense, we would expect to observe a triplet for SnF_2_. However, the four coordination sites are not saturated in SnF_2_, and, thus, there is an exchange with other impurity‐related compounds, such as water. Birchall and Dénès found the same behaviour. They claimed that the missing coupling between ^19^F and ^119^Sn is due to the exchange between different hydrated species of SnF_2_.^[35]^ Our samples contain water since non‐anhydrous [D_6_]DMSO was used as a solvent for the NMR experiments, therefore agreeing with the previously reported experiences. For other species, no splitting occurs since chloride, bromide, and iodide do not have NMR‐active nuclei in significant amounts or with detectable line widths.

This affinity of fluorides towards Sn^IV^ can have several important implications that may reduce Sn^IV^ content in the final film. For instance, the strong preference for Sn^IV^ means that fluorides could complex it as soon as it is generated, whether from O_2_ in the environment or DMSO‐driven oxidation.[^7^, ^8^, ^11^] In fact, one should think if SnF_2_ would be as valuable for other solvents as in DMSO, as fluorides could be critical in sequestrating Sn^IV^ as soon as it is oxidized by this solvent, making it less harmful. The conversion of SnI_4_ into SnF_4_ will also prevent the SnI_4_‐driven degradation pathways recently described by Lanzetta et al.^[36]^ Furthermore, the selective complexation of Sn^IV^ as SnF_4_ may hinder its ability to form any perovskite‐like complex in solution. It has been widely reported for SnF_2_ that this material's excess tends to undergo phase separation.[^22^, ^25^, ^28^] Conclusively, if Sn^IV^ is retained as SnF_4_, it would be challenging to incorporate this form into the perovskite lattice. Instead, it would be displaced to grain boundaries or even removed from the film. As a result, the point defects resulting from incorporated Sn^IV^ in the perovskite lattice can be significantly reduced. To prove this, we compared the different Sn^IV^ species’ ability to coordinate with FAI by analyzing the ^1^H‐NMR of these solutions. Figure S4 shows how all SnI_2_ (FASnI_3_), SnF_4_ and SnI_4_ cause the splitting of the FAI aminic protons, pointing out a certain degree of interaction between the species. However, the signals for all N‐ and C‐attached protons are slightly shielded in the FASnI_3_ solution (where the formation of perovskite adducts in solution occurs), whilst for the case of SnF_4,_ there is no shielding, suggesting that the interaction of SnF_4_ species might have a lower affinity towards perovskite precursors. Moreover, the shift is very pronounced for SnI_4_, which might imply strong coordination with FAI and an increased ability to get incorporated in the perovskite, resulting in adverse consequences for the photovoltaic properties of the films.

**Figure 3:**
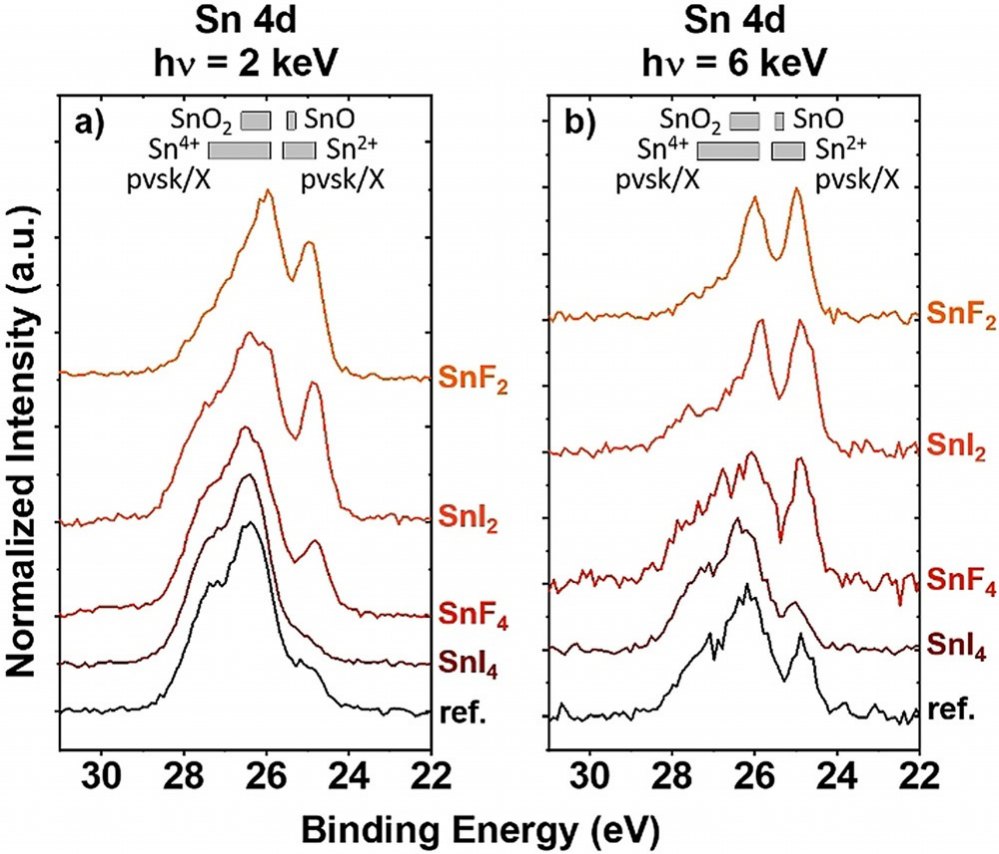
HAXPES spectra of Sn 4d core levels of FASnI_3_ films prepared without (“ref.”) and with 10 mol % of SnF_2_, SnI_2_ (excess), SnF_4_ and SnI_4_, measured using a) 2 keV and b) 6 keV excitation and normalized to maximum intensity (after background subtraction). The used additives are labelled next to the corresponding spectra. The grey‐filled boxes denote the binding energy of Sn 4d_5/2_ of Sn‐based reference compounds reported in the literature.[^30^, ^37^, ^38^, ^39^] “Sn^2+^ pvsk/X” stands for perovskite (tin halide salt) compounds with various ASnX_3_ (SnX_2_) compositions. “Sn^4+^ pvsk/X” stands for perovskite (organotin halide) compounds with various ASnX_6_ (Ph_3_SnX) compositions.

The impact of Sn^IV^ complexation by fluoride in the preparation of FASnI_3_ on the Sn chemical environment in the resulting films was investigated via HAXPES. For that, 10 mol % of SnF_2_, SnI_2_, SnF_4_ and SnI_4_ was deliberately added to FASnI_3_ perovskite precursor solutions. Figure 3 presents HAXPES spectra of the Sn 4d energy region of FASnI_3_ samples prepared with and without various additives, measured with excitation energies of 2 keV and 6 keV, respectively. It is possible to vary the probing depths of the HAXPES measurements using different excitation energies, that is, the 2 keV data is more surface‐sensitive than the 6 keV data (see methods section). The spectra shown in Figure 3 do not exhibit a line shape that resembles a single Sn 4d_5/2_‐4d_3/2_ doublet peak (i.e., with a 3:2=4d_5/2_:4d_3/2_ area ratio and a 4d_5/2_‐4d_3/2_ spin‐orbit separation of ≈1.1 eV);[^30^, ^40^] this is a clear indication that spectral contributions from more than one Sn chemical species are detected. Overall, the Sn 4d spectra suggest substantial contributions in the binding energy (BE) regions (24.9±0.1) eV and (26.2±0.2) eV, corresponding to values reported in the literature for Sn 4d_5/2_ of Sn‐based perovskite/halide and oxide reference compounds with Sn being in Sn^2+^ and Sn^4+^ environments, respectively.[^30^, ^37^, ^38^, ^39^] Because the Sn 4d BE values of Sn^2+^ (SnO) and Sn^4+^ (SnO_2_) oxide compounds are energetically overlapping with the respective Sn environments expected to be present in the sample set (i.e., FASnI_3_ and the different Sn‐based additives) and O‐related lines were detected in the HAXPES measurements, the Sn 4d spectra in Figure 3 likely contain Sn oxide derived spectral features and thus consist of more than two doublet peaks. Comparing the spectra in Figure 3 a with the spectra in Figure 3 b demonstrates that the high BE Sn^4+^‐related features are more prominent in the 2 keV measurements than in the 6 keV, which indicates an increased prevalence of the Sn^4+^ related species (in the form of SnO_2_ or SnX_4_) near the surface of samples than deeper within their bulk. However, significant differences in the line shape of the spectra for a given excitation set reveal pronounced changes in the Sn chemical environment of the investigated samples concerning the presence/absence and kind of additives during processing. The spectra of samples with additives containing Sn^2+^ or F^−^ display a significant increase in the low BE Sn^2+^‐related signal. This finding seems to be in line with the NMR results described above, that capturing Sn^IV^ in the form of fluorinated species prevents its incorporation in the films. However, the observed variability of properties at the surface of the samples associated with handling conditions of the Sn‐based perovskite samples (as has been already reported,^[30]^ and further discussed in Supporting Information, see Figure S5) prevents the present interpretation of the HAXPES results from reaching further conclusions on the impact of individual additives with statistical certainty.

As depicted previously in Figure 2 a, we attribute the color change of FASnI_3_ in DMSO to the change in solution properties and not to the Sn^IV^ content. Nevertheless, it is crucial to understand the underlying reasons that led to this change, as we expect it to exhibit a decisive influence on the crystallization dynamics.

**Figure 4:**
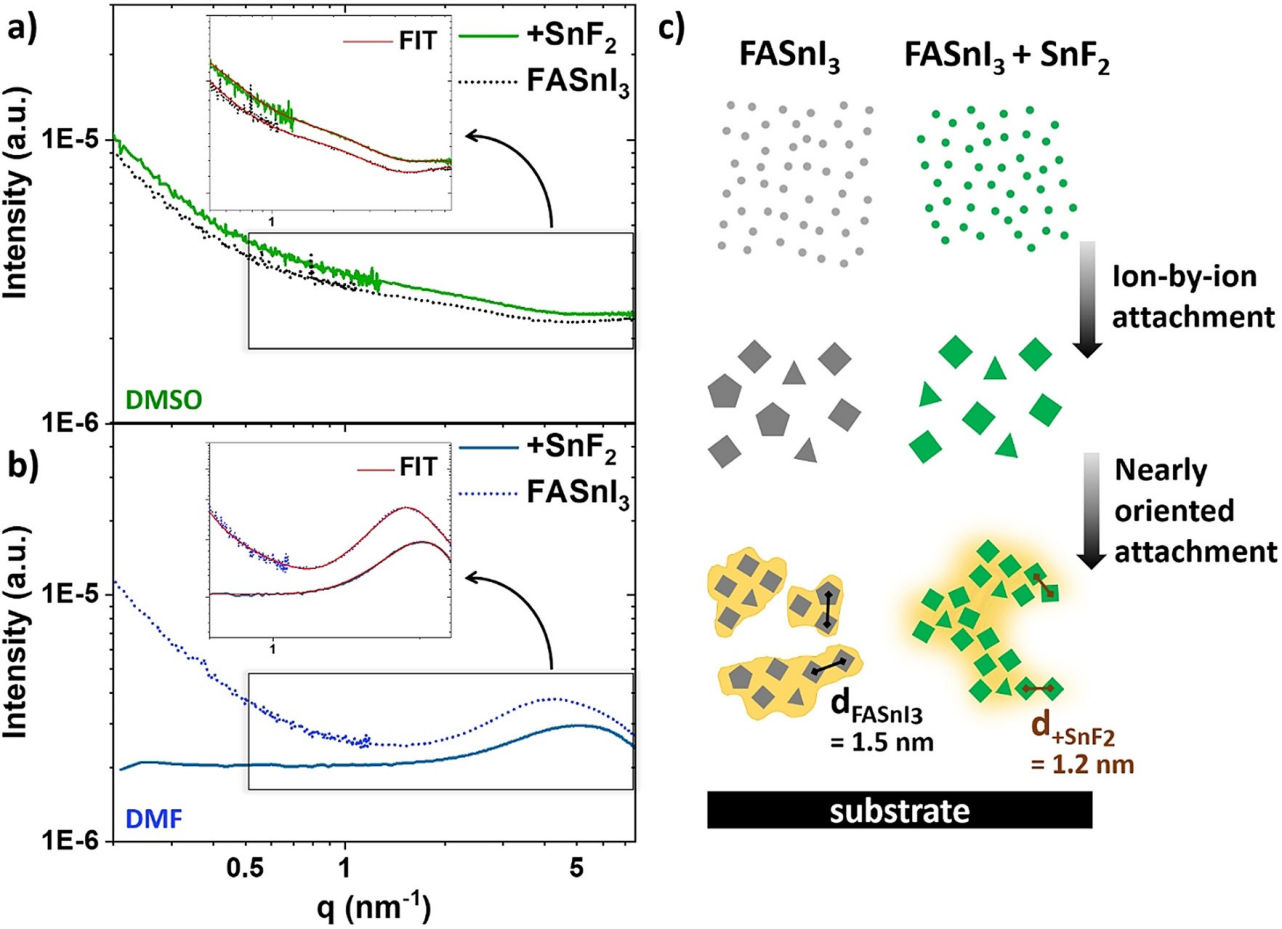
SAXS performed on different FASnI_3_ precursor solutions. SAXS curves of FASnI_3_ (dotted line) compared to FASnI_3_ with SnF_2_ addition (solid line) in a) DMSO and b) DMF, as well as the corresponding fit given in red in the magnified representations. c) Proposed nucleation and growth mechanism in FASnI_3_ precursor solution affected by SnF_2_ addition, where the different forms (triangle, quadrangle, pentagon) should schematically describe the potential variety of subunits.

Here, we perform transmission small‐angle X‐ray scattering (SAXS) to reveal the effect of SnF_2_ on the perovskite precursors in solution. Thereby, a proposed nucleation mechanism indicates that the use of SnF_2_ promotes homogeneously distributed growth, yielding improvements in the overall crystal quality. Using the SAXS instrument at BESSY II at X‐ray energies of 8 keV and 10 keV (Δ*E*/*E*=2×10^−4^), we cover a *q*‐range from 0.05 to 8.5 nm^−1^ (size range: 209.4–0.74 nm). Figure 4 a compares the SAXS scattering curve of a plain FASnI_3_ solution in DMSO with FASnI_3_ containing SnF_2_. At first sight, the comparison does not show significant variations.

Nevertheless, by applying a model fit using the software SASfit©,^[41]^ which offers several different form and structure factors describing various shapes of particles and their interaction, small changes regarding the particle interplay in the high *q*‐region can be observed. Here, however, interpretation requires particular precaution since we are already in the proximity of interatomic distances. The general behaviour of the initial perovskite precursor stage is highly dependent on the specific solvent environment. Literature shows that solvents with a lower donating number interact weakly, whereas stronger donating solvents interact strongly with the metal of a perovskite precursor solution.^[42]^ Therefore, stronger donating solvents tend to hinder the iodide coordination of the metal. Since DMSO is known to be strongly donating and hence decelerates the perovskite crystallization process, we here include *N*,*N*‐dimethylformamide (DMF) with lower donating effect to investigate further the possible influence of SnF_2_ on the early stages of crystallization. Figure S6 shows the same effect on the color of SnF_2_ in DMF as in DMSO, as proof that the same visual transformation occurred.

The evolution of a maximum in the SAXS scattering curve of FASnI_3_ in DMF given in Figure 4 b shows a clear difference compared to the scattering curve of pure FASnI_3_ in DMSO. The maximum emerges based on a dominant structure factor, which evolves due to particle interaction. The mean spacing *d* between the mass centers of the individual interacting particles can be calculated as discussed by Raghuwanshi et al. using the magnitude of *q* at the peak maximum.^[43]^ In the plain FASnI_3_ solution in DMF, this results in a mean spacing *d* of approximately 1.5 nm. Adding SnF_2_ to the solution leads to a shift of this peak maximum to higher *q* and, consequently, lower mean *d* spacing of 1.2 nm. Besides the shift of the maximum, also the slope at lower *q*‐values disappears. The shallow negative slope for both DMF and DMSO solutions gives rise to the presence of larger structures with a broad size distribution (>100 nm) in the solution. We propose that the larger sizes represent aggregates consisting of small interacting subunits formed by ion‐to‐ion attachment. We assign these subunits to particles or clusters in an average dimension of 0.4 nm observed in all scattering curves. In the DMF case, we assume that these aggregates form by nearly oriented attachment, as described in the non‐classical nucleation theory being pre‐ordered arrangements (Figure 4 c).[^44^, ^45^, ^46^] The well‐pronounced structure factor peak can evidence this, showing the recurring distance *d* between subunits, representing the average distance between the mass centers of the units and could thus be considered the tin‐to‐tin distance due to the high electron density of tin. A specific recurring distance *d* can also be noticed in the case of SnF_2_ addition. However, there is no negative slope at low *q*‐values assigned to larger higher‐level structures. Therefore, we conclude that the total size distribution generally appears to be more homogeneous; the nearly oriented attachment with the recurring distance *d* of 1.2 nm might be considerably more extensive than in the plain FASnI_3_ solution or even of infinite size. Additionally, we performed several runs for every sample to prove no damage caused by the beam (Figure S7).

Pre‐ordered arrangements of subunits set the starting point for the further crystallization of a thin film on a substrate. The broad size distribution of comparable smaller aggregates might result in films including unordered pores or pinholes because solvent evaporation leaves holes between the pre‐ordered totals. Instead, the more uniform size distribution due to a larger oriented attachment of the subunits supports homogeneously distributed crystal growth, a suitable substrate coverage and improved film morphology, precisely what is observed by SnF_2_ addition in literature.[^22^, ^25^, ^28^] With the premise of the already advanced aggregation of elements in the DMF solvent, a similar mechanism during the late stages of enhanced crystallization may be expected for the case of DMSO. By applying pressure via spin coating and solvent evaporation, the concentration of FASnI_3_ in the solution increases. Following the evolution of SAXS scattering curves for FASnI_3_ concentration series in DMF and DMSO, it suggests that a structure factor maximum is formed at higher concentrations in the case of DMSO comparable to the DMF solution (Figure S8). In this sense, the observed behaviour for DMF can be extrapolated to a more advanced stage of precursor formation for the DMSO precursor solution. Therefore, SnF_2_ as an additive leads to an in total more homogeneous crystallization of the tin halide perovskite thin film, and thus to a better morphology.

Regarding the change in solution color caused by SnF_2_ addition, we speculate that fluoride modifies the coordination level of tin centers by iodide ions, hindering the formation of colorful, highly coordinated [SnI_*x*_]^2−*x*^ units. The fact that better morphology films are obtained through the pale yellow, SnF_2_‐containing solution points out that, with solution color as an indication, the properties of the existing formations in solution critically influence the crystallization dynamics of tin halide perovskites. This feature is currently underexplored for these materials and proves to be much more complex and sensitive than for their lead analogues due to its quite restricted processing conditions.

Other SnX_2_ (X=Cl, Br, I) and their influence on perovskite properties are also frequently discussed in the literature.[^16^, ^17^, ^47^] We further performed SAXS on FASnI_3_ precursor solutions according to SnX_2_ addition, given in Figure S9, to compare their respective functionality to the SnF_2_ addition. Similar to the scattering curves shown in Figure 4 a, no significant influence or difference between different X can be noted. However, it should not be ruled out that they could have the same behavior difference as SnF_2_ in other solvents like DMSO and DMF. This confirms the need to investigate the strong dependence of additives and compositions used for tin halide perovskites. Finally, a scattering curve for the presence of Sn^IV^ is given in the inset window in Figure S9, for which we measured an aged sample of FASnI_3_. The effect of temperature‐induced degradation of DMSO solutions on its properties seems relevant, confirming that we did not influence unexpected Sn^IV^ content in FASnI_3_ with or without SnX_2_.

**Figure 5:**
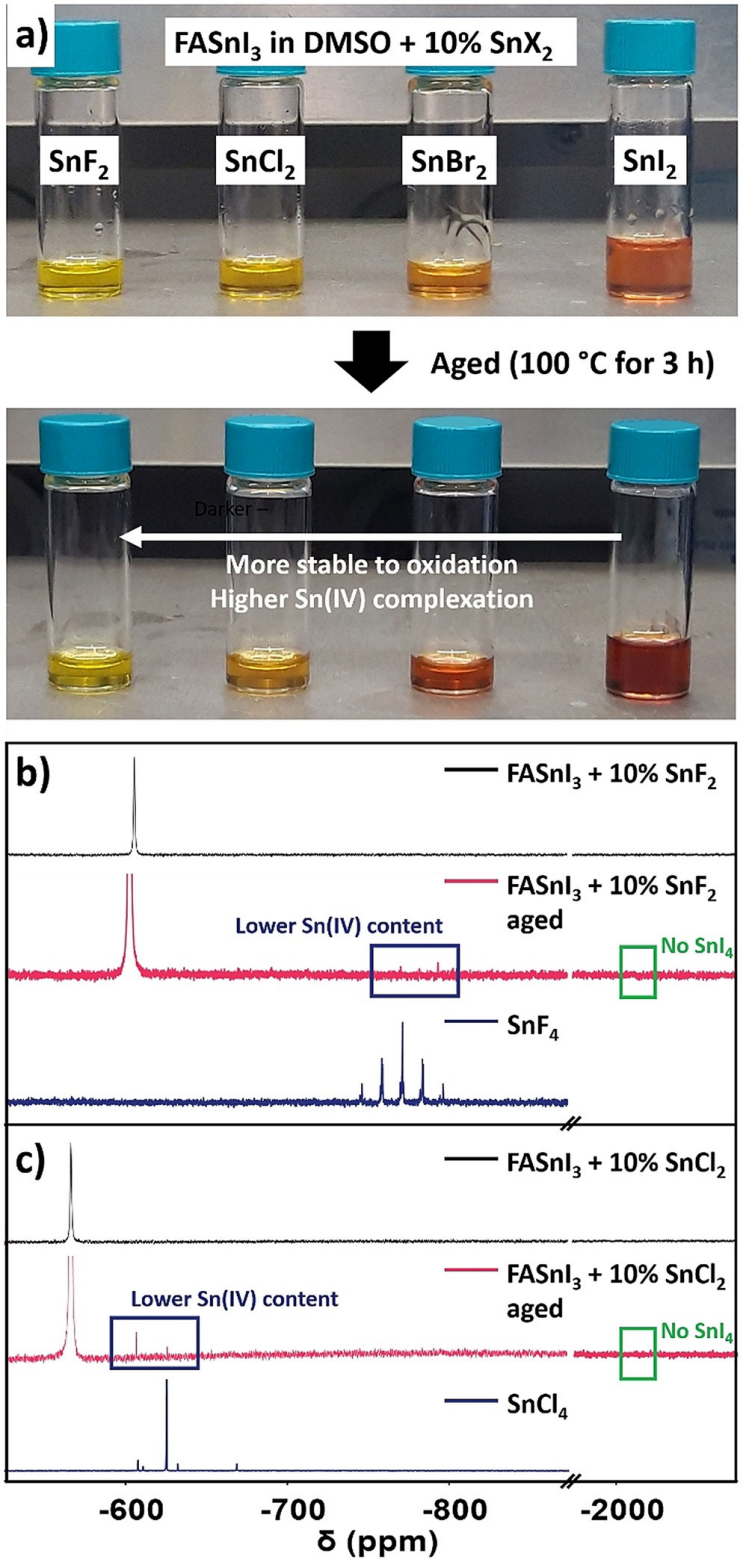
a) Fresh and aged solutions of FASnI_3_ in DMSO with 10 % SnF_2_, SnCl_2_, SnBr_2_ and SnI_2_. An arrow points out the difference in darkening as an effect of the ageing treatment. ^119^Sn‐NMR spectra of b) 10 % SnF_2_‐ and c) 10 % SnCl_2_‐containing FASnI_3_ solution before and after being aged. SnF_4_ and SnCl_4_ solutions spectra are added as indicative of the species formed under the ageing process.

Although SAXS detected no difference for the different SnX_2_ additives in DMSO solutions, they still caused a color change in a clear trend (Figure 5 a). Both SnF_2_ and SnCl_2_ led to a similar yellow coloration of FASnI_3_ solution, while SnBr_2_ affected it mildly more. This trend could mean that the colorful, highly coordinated [SnI_*x*_]^2−*x*^ iodostannates were hindered more strongly as the halide X is a harder Lewis base. The fresh solutions were analyzed by ^119^Sn‐NMR (Figure S10), although no notable difference was found except for the shielding effect from SnF_2_, already discussed in Figure 2. In this sense, SnF_2_ is the only SnX_2_ additive shifting the FASnI_3_ signal upfield, while SnCl_2_ and SnBr_2_ go slightly in the opposite direction. This effect correlates well with the chemical shifts in the different SnX_2_‐pure solutions in DMSO (Figure S11), except for the SnI_2_ case. Hence, the final Sn^II^ signal position might be an average value of all Sn^II^ species. After heating the solutions, we observed that the solution's darkening was negligible for chloride‐ and fluoride‐containing solutions, which could be due to both lower Sn^II^ oxidation and more efficient Sn^IV^ complexation by these anions, as explained in Figure 2. The heated solutions were measured by ^119^Sn‐NMR (Figure 5 b), showing that the content of Sn^IV^, all in the form of SnF_4_, had been significantly reduced in comparison to SnF_2_‐free heated solution (Figure 2 c). We hypothesize that fluoride could affect DMSO and Sn^II^ environments, maybe through the modulation of [SnI_*x*_]^2−*x*^ adducts, making these two species less eager to undergo a redox reaction. Also, we observed that SnCl_2_ addition had the same effect as SnF_2_, leading to both reduction of the oxidation and the selective complexation of Sn^IV^ through the formation of SnCl_4_ (Figure 5 c). These results prove that hard Lewis bases like chloride and fluoride can block the formation of Sn^IV^ in the solution and their introduction into the perovskite film through two different mechanisms: complexation of Sn^IV^ and antioxidative character. Our findings agree with previous reports on reducing Sn^IV^ by the addition of SnF_2_[^23^, ^28^, ^30^] or SnCl_2_[^16^, ^17^] and suggest that many of the other additives employed in literature for tin halide perovskites may work in the same fashion.

To confirm that fluoride was responsible for these changes in solution, we prepared FASnI_3_ solutions containing other fluoride‐based compounds. Unfortunately, other common species (i.e. CsF and NaF) had limited solubility in common solvents. Therefore we had to saturate the FASnI_3_ solution below a 5 % molar ratio (Figure S12). Even though the concentration was lower than for SnF_2_, we observed the exact change in color from orange to pale yellow, potentially affecting perovskite subunits in solution in the same fashion. Similarly, these solutions experienced no darkening of solutions aged at 100 °C for 3 h in different conditions, proving the complexation of Sn^IV^ in the form of SnF_4_. Even though these particular additives may not be directly implementable due to the strong influence that Cs^+^ and Na^+^ cations can have in the perovskite solar cells processing and performance, these results confirm the universality of the working principle for fluoride‐based compounds. Furthermore, they suggest that SnF_2_ additive may be eventually replaceable by other fluoride‐based species if applied in the right conditions.

To complete the study, we wanted to investigate how these changes in perovskite solution properties affect the thin film formation and the corresponding solar cells performance. We fabricated pristine FASnI_3_ films and with 10 % of the excess of FAI, SnI_2_, SnBr_2_, SnCl_2_ and SnF_2_. Excess FAI was tried to study both stoichiometry sides of FASnI_3_. While there was no notable change in the X‐ray diffraction patterns (Figure S13), the scanning electron microscopy (SEM) results offered some differences among samples (Figure S14). The sample with 10 % SnI_2_ excess was the only one showing a high density of extensive pinholes. In contrast, the film with 10 % SnF_2_ was the most homogeneous one, free of pinholes and other minor irregularities that are present in the rest of the films, agreeing with previous papers that used SnF_2_ on its beneficial effect on morphology.[^22^, ^25^, ^28^]

There is an evident change in the resulting grain size with the changing halide element (Figure S15). Fluoride led to the smallest average grain size (568 nm) compared to chloride (629 nm) and bromide (623 nm). These results are orthogonal to those reported previously, where fluoride^[25]^ and chloride^[17]^ were said to increase the perovskite crystals’ grain size. However, tin halide perovskites’ sensitive nature implies that significant changes can be expected from minor modifications in the perovskite composition or processing. Therefore, the effect of halides introduction can vary from study to study. Also, stoichiometry in pure FASnI_3_ perovskite strongly affects the pinhole density and the average grain size. The largest size was found for equimolar FASnI_3_ (695 nm), which went down when increasing or decreasing the SnI_2_ ratio (647 and 568 nm, respectively). Moreover, SnF_2_ addition shows an impact on the size distribution itself, which is significantly narrowed to plain FASnI_3_ thin film. These observations agree with the results by SAXS, assuming that a uniformly nearly oriented attachment in solution leads to a more homogeneous distribution of the grain sizes in the film.

We then used these films for solar cells fabrication (more details in the Supporting Information) to investigate any possible trend between additives and performance. In this sense, adding a small portion of tin halides to FASnI_3_ solutions seems beneficial for the device performance, showing some positive trend when moving to lower size halides (Figure S16). Though it appears that smaller halides—harder Lewis bases—work better by having a more decisive influence in the processing, chloride was the exception. Even though NMR and SAXS found SnCl_2_ to have very similar behavior to SnF_2_, the resulting devices yielded no efficiency, suggesting that chloride brings other factors into play. Previous works point out how chloride can be incorporated in the lattice and its tendency to form massive aggregates,[^16^, ^17^] making its application not as trivial as SnF_2_ and requiring a more careful optimization. We suspect SnCl_2_ could mimic SnF_2_ to some extent in these solutions if the processing conditions are adjusted accordingly. It is also worth noting the slight improvement in efficiency produced just by using a 1,1:1 SnI_2_:FAI stoichiometry (i.e. 10 % SnI_2_ excess), despite the content of irregularly sized pores (Figure S14). This matches well with the results in previous works,[^11^, ^32^] proving the importance of providing a Sn‐rich environment in the film.

## Conclusion

SnF_2_ is a widely used additive for tin and lead/tin halide perovskites, systematically showing the same beneficial effects in all reported studies: perovskite films with lower Sn^IV^ content and improved morphology. We uncovered the different roles of fluoride in SnF_2_ on Sn^IV^ complexation and colloidal arrangement in the precursor solution. By studying the fluoride chemistry in perovskite solutions and films with different complementary techniques, we demonstrated that the fluoride in SnF_2_ has a critical role in reducing Sn^IV^ content in the precursor solution and the final perovskite film. We showed by NMR the selective complexation of Sn^IV^ in the form of SnF_4_, which HAXPES revealed to have a lower tendency to get introduced in the film than SnI_4_. Moreover, we showed how the introduction of SnF_2_ in perovskite solutions increases their stability against the oxidation caused by DMSO. This antioxidative character was also found for SnCl_2_, meaning that many other reported additives for tin halide perovskites may also block Sn^II^ oxidation by simple tuning of solution properties. Apart from reducing Sn^IV^ content in the thin film, SAXS measurements on the related precursor solutions evidenced that fluoride alters the essential formation of pre‐organized perovskite clusters. We identified an advanced colloidal arrangement in DMF compared to the DMSO solutions that are notably influenced by the addition of SnF_2_. We assigned this arrangement to an advanced nucleation process in DMF compared to DMSO. Finally, based on our findings, we proposed a nucleation mechanism that occurs in solution and is affected by the SnF_2_ addition resulting in improved overall crystal quality. In this sense, the effect of SnF_2_ on the film processing will be strongly determined by the environment in which it is applied (i.e. solvent, perovskite composition). Consequently, there is an immediate need to fundamentally understand and optimize solution properties, their processing and studying the effect of additives. As we are doing in this study with the example of SnF_2_ as pioneering work and impulse for further research. Overall, we presented a complete comprehensive picture of the working mechanism of SnF_2_ in tin halide perovskites processing and provided the community with the guidelines for finding new additives with specific chemical properties to selectively complex Sn^IV^ species and regulate the crystallization.

## Conflict of interest

The authors declare no conflict of interest.

## Supporting information

As a service to our authors and readers, this journal provides supporting information supplied by the authors. Such materials are peer reviewed and may be re‐organized for online delivery, but are not copy‐edited or typeset. Technical support issues arising from supporting information (other than missing files) should be addressed to the authors.

Supporting Information
