# Supplementary material for: Fluoride Chemistry in Tin Halide Perovskites
Source: Angew Chem Int Ed Engl. 2021 Jul 24;60(39):21583–91. doi: 10.1002/anie.202107599 (PMC8518082; doi:10.1002/anie.202107599)
Supplement: Supplementary file 1 — Supporting Information [file ANIE-60-21583-s001.pdf]

Angewandte Chemie
Eine Zeitschrift der Gesellschaft Deutscher Chemiker

Supporting Information

Fluoride Chemistry in Tin Halide Perovskites

Jorge Pascual[+], Marion Flatken[+], Roberto Félix, Guixiang Li, Silver-Hamill Turren-Cruz, Mahmoud H. Aldamasy, Claudia Hartmann, Meng Li, Diego Di Girolamo, Giuseppe Nasti, Elif Hüsam, Regan G. Wilks, André Dallmann, Marcus Bär, Armin Hoell, and Antonio Abate**

anie_202107599_sm_miscellaneous_information.pdf

Supporting Information

Experimental Procedures

Materials

SnI_2, $SnBr_2$ and $SnCl_2$ were purchased from TCI and formamidinium iodide (FAI) from Dyenamo. Dimethyl sulfoxide (DMSO), SnF_2 (99 %), SnF_4, SnI_4 (99.999 %), $SnCl_4$ (98 %), C_{60} (99.5 %) and chlorobenzene were purchased from Sigma-Aldrich. Poly(3,4-ethylenedioxythiophene: poly(styrenesulfonate) (PEDOT:PSS) Clevios P VP Al 4083 was obtained from Heraeus Deutschland GmbH & Co. KG. Bathocuproine (BCP, 99.9 %) was acquired from Ossila. Silver shots (Ag, 99.95 % purity) were purchased from Alfa Aesar.

Solution preparation

1 M $FASnI_3$ perovskite precursor solutions were prepared by adding in 1:1 stoichiometry the corresponding volume to FAI powder from a stock 1.2 M solution of SnI_2 in DMSO, and DMF as well for SAXS experiments. SnX_2-containing (X = F, Cl, Br, I) solutions were prepared by adding the corresponding volume of the 1 M $FASnI_3$ solution to the SnX_2 powder, according to its molar percentage (i.e. 5, 10 and 20 % for F and 10 % for Cl, Br and I). Illustrative CsF- and NaF-containing 1 M $FASnI_3$ solutions in DMSO were prepared by saturating the precursor solution with the corresponding powder. The rest of the solutions were prepared by simply dissolving the powders in DMSO close to their saturation level (i.e. SnF_2, $SnCl_2$, $SnBr_2$, SnF_4, $SnCl_4$, $SnBr_4$ and SnI_4 at 0.5 M). SnF_4 solution was heated at 80 °C for 1 min to promote dissolution. If presenting precipitates, solutions were filtered prior to their characterization.

Thermal Ageing

The thermal stress of the solution was performed at 100 °C for 3h. We took pictures before and after the ageing treatment.

Device fabrication

Patterned indium tin oxide (ITO) glass substrates (25x25 mm, resistivity = 15 Ω sq^{-1}, nominal ITO thickness = 150 nm, patterned by Automatic Research GmbH) were cleaned sequentially for 15 min with a 2 % Mucasol solution in water (Schülke), water, Acetone, and Isopropanol at ~ 40 °C in an ultrasonic bath. After the substrates were dried with N_2 flow, they were treated in a UV-ozone cleaner for 30 min. A PEDOT:PSS solution (180 µL) was spin-coated on FTO substrates at 4500 rpm for 45 s, followed by annealing at 150 °C for 10 min in air. Before use, PEDOT:PSS dispersion was filtered with 0.45 µm PVDF filter. The substrates were transferred into a N_2-filled glovebox, and from here the rest of the processing and measuring was carried out in inert conditions, avoiding all possible contact of the samples and devices with air. 100 µL of perovskite precursor were spin-coated on ITO/PEDOT:PSS substrates at 5000 rpm for 70 s with a ramp of 1000 rpm s^{-1}. After 50 s, 200 µL of chlorobenzene as antisolvent were dripped onto the perovskite film. The substrates were quickly transferred to a hot plate with a program controller for 10 min at 100 °C. Finally, C_{60} (23 nm), BCP (8 nm) and Ag (100 nm) were thermally evaporated and deposited sequentially on ITO/PEDOT:PSS/perovskite layer in a MBRAUN ProVap 3G at a base pressure of 10^{-6} mbar. Ag was thermally evaporated through a shadow mask, where the overlap of the ITO with the Ag stripe defined an active area of 0.16 cm^2.

Current Density−Voltage Measurement

The *J-V* measurement is done with 1 sun equivalent illumination from an Oriel LCS100 class ABB solar simulator in an inert atmosphere without cooling. The lamp is calibrated with a Si 1 cm^2 diode certified by Fraunhofer ISE.

Scanning electron microscopy (SEM)

SEM images were recorded using a Hitachi S4100 or ZEISS Auriga 60 at 5 kV acceleration voltage and 10k and 30k magnification.

Grazing Incident X-ray Diffraction

The measurements were conducted in the Bruker AXS D8 ADVANCE X-ray diffractometer (multipurpose diffractometer) and Bruker AXS DIFFRAC.SUITE software, using grazing incidence geometry. Samples were kept in nitrogen atmosphere using half-spherical domes to avoid air oxidation. XRD pattern is collected with a step size of 0.01 degree, for 2 seconds each step.

Small Angle X-Ray Scattering:

The SAXS measurement was performed using synchrotron radiation at the four crystal monochromator X-ray beamline in the laboratory of PTB (Physikalisch-Technische Bundesanstalt) at BESSYII.[1] The SAXS instrument of Helmholtz-Zentrum Berlin (HZB) contains an adjustable about 3 m long support structure with a long edge-welded bellow system to enable the changing of the sample to detector distance.[2] The 2D scattering images were collected by a windowless Dectris 1M PILATUS2 in-vacuum hybrid-pixel detector. The measurements were carried out at two different distances (0.8 m and 3.7 m) at photon energies of 10 keV and 8 keV, respectively. X-ray energies and sample to detector distances are chosen to get the maximum possible q-range and with respect to the low comparably sample transmission. Thus, a *q*-range from 0.05 nm^{-1} to 8.5 nm^{-1} was covered (size range of 125.6 to 0.74 nm in real space). The precursor solutions were measured with an acquisition time of 600 s with three repetitions for the short distance and long distance to achieve good quality data and to monitor unwanted but possible changes of the specimen over time (which can therefore be safely excluded). Due to the low transmittance of the lead containing precursor solutions, especially thin (0.1 mm), rectangular borosilicate cuvettes (with a wall thickness of 0.1 mm) purchased from CM Scientific, UK were used. Using a low-scatter pinhole of germanium 500 µm in diameter, the total flux on the sample has been determined for each energy. For the long distance (3.7 m) at 8 keV, we indicated 4.8×10^9 photons/s and 2.6×10^9 photons/s for the short distance (0.8 m) at 10 keV.

Data reduction and radial averaging & fitting: For data reduction as well as for the radial averaging to the 1D scattering pattern the BerSAS software was used, an advanced version of the BerSAS software[3] applicable for SAXS and SANS. All SAXS curves of the investigated samples were fitted with the program SASfit[4]. In order to get a general idea of the order of magnitude a structure model that includes a spherical form factor and a hard-sphere structure factor was chosen to fit all sample scattering curves. The herein investigated particles were considered as hard spheres. Thereby, these particles are assumed as incompressible resulting in fixed radii for each particle and an infinite repulsive force at a certain interparticular separation. The hard spheres model neglects attractive forces but describes fairly well a various number of colloids in organic solvents.[5-7] We used the monodisperse Percus-Yevick approximation for hard spheres to fit the interaction of the analyzed colloidal particles.[8,9]

Hard X-ray photoelectron spectroscopy:

Hard X-ray photoelectron spectroscopy (HAXPES) measurements were carried out at the HiKE endstation located at the BESSY II KMC-1 beamline at Helmholtz-Zentrum Berlin (HZB).[10,11] The HiKE endstation is equipped with a Scienta R4000 electron analyzer and excitation energies of 2003 eV (referred to as “2 keV”) and 6009 eV (referred to as “6 keV”) were employed, using the 1st and 3rd orders of the Si(111) crystal pair of the double crystal monochromator. To prevent beam damage effects on the samples, measurements were conducted with a beam-attenuating (100 □m) Be filter,[11] which reduces the photon flux of the 1st order light to 25 % of the original while transmitting >90 % of the 3rd order photons. The binding energy (BE) scales of the HAXPES measurements were calibrated by measuring the Au 4f energy region of a clean Au foil in electrical contact with the (grounded) sample and setting the BE of the Au $4f_{7/2}$ line to 84.0 eV. The pressure in the endstation’s analysis chamber during the HAXPES measurements remained <1 x 10^{-8} mbar. To prevent/minimize exposure to ambient conditions, samples were mounted and sealed inside a N_2-filled glovebox for transport prior to introduction into the HiKE endstation system. The HAXPES probing depth is an exponential distribution governed by the inelastic mean free path (IMFP) of the probed photoelectrons. Regardless of excitation energy, the strongest spectral contribution comes from the surface of the sample, and with 63 % (i.e., 1/e) of the signal coming from within the first IMFP. For the Sn 4d shallow core levels, measurements using 2 and 6 keV have inelastic mean free path (IMFP) values of ≈4 nm and ≈10 nm, respectively.[12,13] Probing the Sn 4d shallow core levels, which have a narrower line width than the more frequently investigated Sn 3d core levels, was preferred, as spectral contributions of Sn-related chemical species showing only small differences in chemical shift can be better resolved.

NMR:

Liquid-state ^{119}Sn-NMR

The spectra were all acquired on a Bruker AVII 400 MHz or Bruker AVIII 500 MHz equipped with room-temperature TBO or BBO-probe heads respectively. Typically, a sweep width of 504.3 ppm was used and 64k points were acquired, resulting in a total acquisition time of 435.81 ms. The center frequency had to be adjusted from sample to sample in order to detect the desired signal, therefore, on new samples, a full scan of the possible shift range was acquired (1000 to -3000ppm). We used a 30 ° pulse in order to minimize the recycle delay down to 2 s. The number of scans thus ranged from 128 for very concentrated samples to 16k scans for very dilute samples.

The spectra presented differences in the signal-to-noise ratio, thus the higher noise in some of the spectral baselines, particularly when zooming in to show the SnI_4 signal more clearly. All existing signals in all spectra were identified and pointed out.

Liquid-state ^{1}H-NMR

All other spectra were measured with standardized parameter sets from Bruker Topspin version 2.1 (AV400) and 3.0 (AV500). For non-deuterated solvents, a borosilicate capillary containing DMSO-d6 was introduced in the NMR tube for allowing the locking. The capillaries were 1.5 mm wide, 80 mm long and 0.01 mm wall-thick and were purchased from WJM-Glas/Müller GmbH.

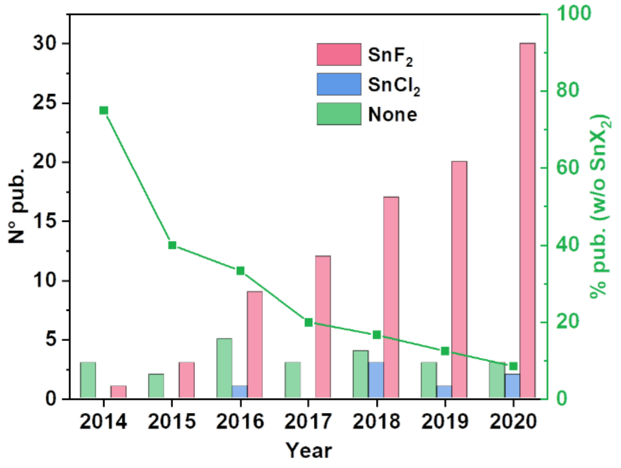

Figure S1. Number of scientific publications per year using SnF_2, $SnCl_2$ or none of them. The green line, linked to the right-side vertical axis, illustrates the decline in the relative number of studies based on strategies that avoid the use of SnF_2 or $SnCl_2$.

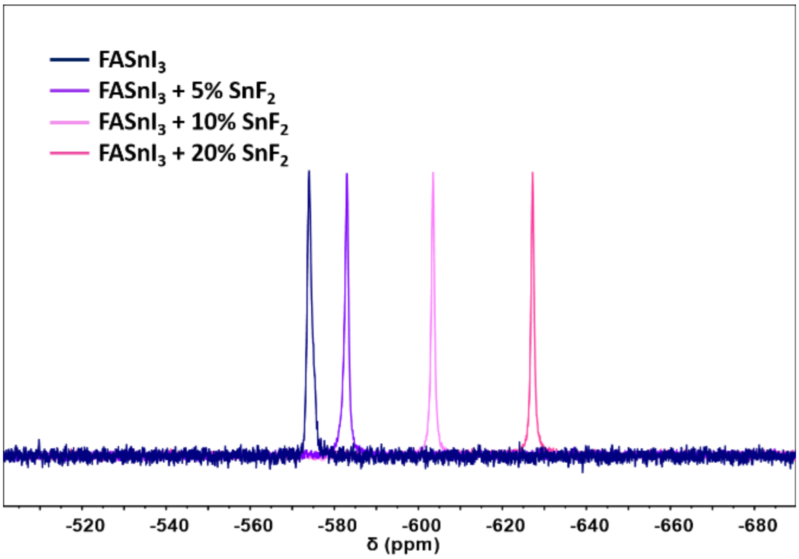

Figure S2. ^{119}Sn-NMR of 1 M $FASnI_3$ perovskite precursor solution in DMSO and with different molar concentrations of SnF_2.

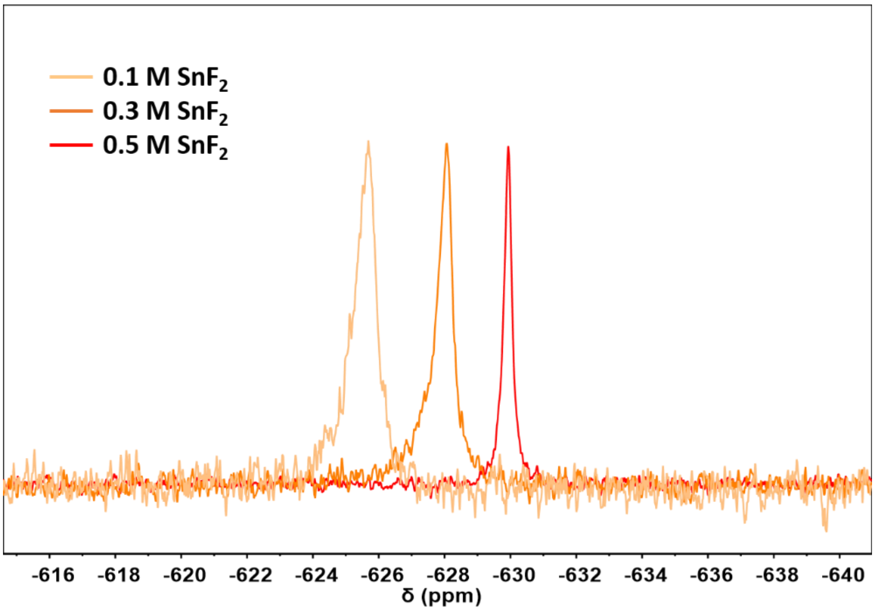

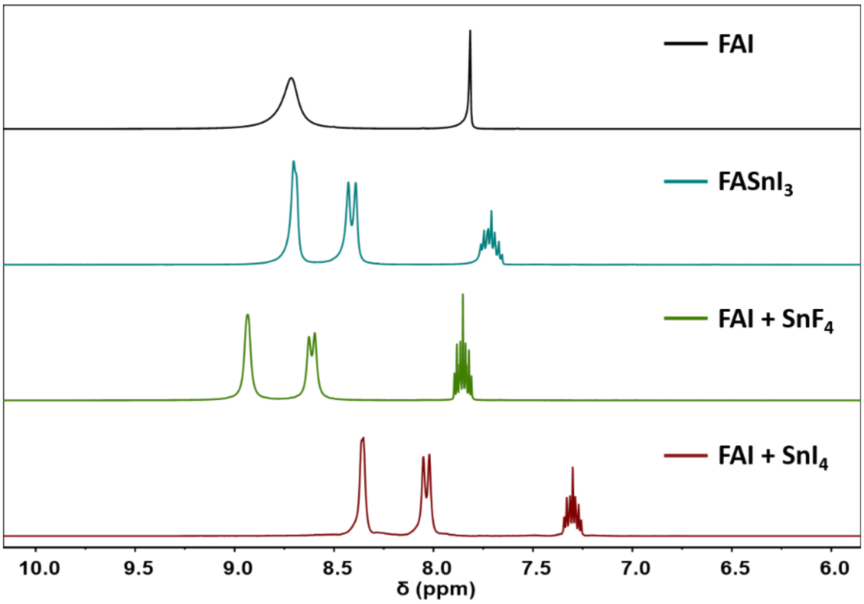

Figure S3. ^{119}Sn-NMR of [illegible] SnF_2 in different concentrations in DMSO. [illegible] More in detail about these and other solutions [illegible] preparation, SnI_2 was dissolved at 1 M, while [illegible] SnI_4, SnF_2 and SnF_4 were saturated (up to 0.5 M). [illegible] However, as we show in this figure this should not [illegible] affect the comparison, as SnF_2 signal barely shifts [illegible] with [illegible] solution concentration. SnF_4 is [illegible] more restricted to interactions with the [illegible] solvent due to the higher complexation degree by [illegible] fluoride ions and therefore would show even lower [illegible] signal shift, as it happened with SnI_4 in our previous [illegible] work.[14]

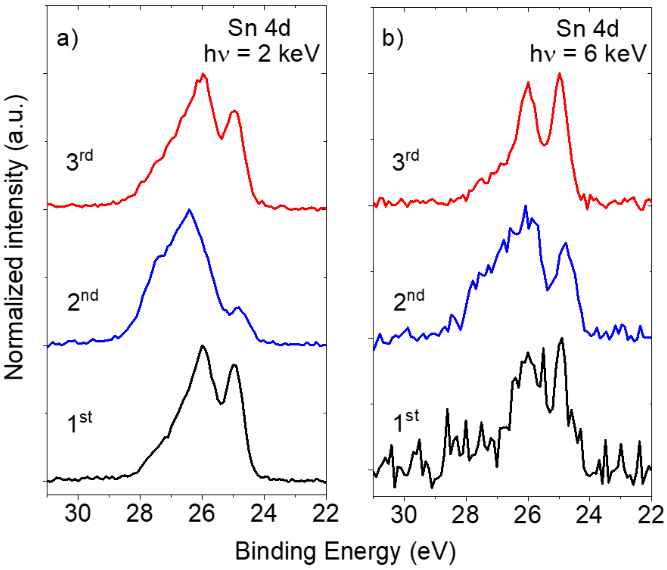

Figure S4. ^{1}H-NMR of FAI, $FASnI_3$, $FAI+SnF_4$ and $FAI+SnI_4$ solutions in DMSO at 1:1 stoichiometry. The range was restricted to 6-10 ppm for better resolution of the FAI protons.

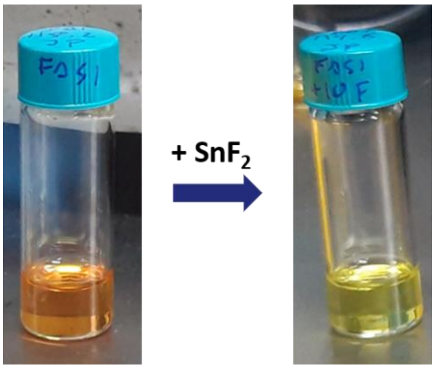

Figure S5. Hard X-ray photoelectron spectroscopy (HAXPES) spectra of Sn 4d core levels of three nominal identical $FASnI_3$ films prepared with 10 mol% SnF_2, measured using a) 2 keV and b) 6 keV excitations and normalized to maximum intensity (after background subtraction). The samples were measured on separate dates, in the order denoted by the labels.

Figures S5a and b displays HAXPES spectra of Sn 4d core levels of three $FASnI_3$ films prepared with 10 mol% SnF_2 (using the same recipe) measured using 2 keV and 6 keV, respectively. The three nominally identical samples were prepared and measured on separate dates but following the same procedures and parameters. Differences in Sn chemical environment, which can be specially observed for the 2nd sample, indicate variations in sample properties despite an identical sample preparation process, the reason for which is likely due to a fluke in the conditions in one (or more) of the sample production (e.g., fluctuations in precursors, glovebox conditions, etc.)/handling (e.g., non-optimal selection of sample area to be cut and mounted for measurement)/transporting (e.g., improper sealing)/measurement (e.g., fluctuation in characterization vacuum conditions, duration between sample preparation and measurement, etc.) steps. However, these observed spectral variations should be considered when examining the Sn 4d spectra shown in the main manuscript (see Figure 3), as they are in most cases more pronounced than the differences between individual spectra as a result of the different additives. Therefore, reaching statistically-relevant outcomes, beyond the conservative conclusions presented in the main text, on the impact of individual additives would require measuring multiple sets of this sample series, a task beyond the scope of the present work.

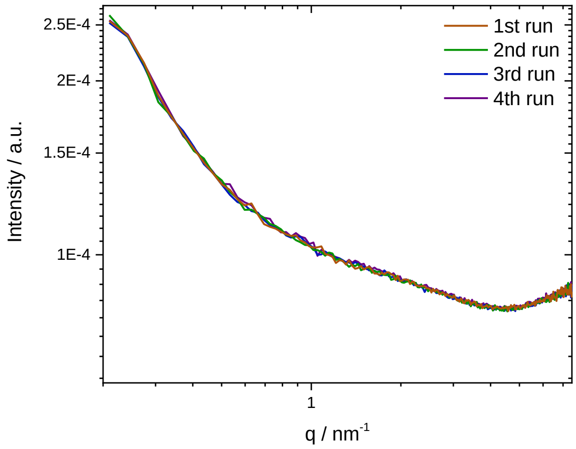

Figure S6. Picture of fresh 1 M $FASnI_3$ solution in DMF (left) and after addition of 10 % SnF_2 (right).

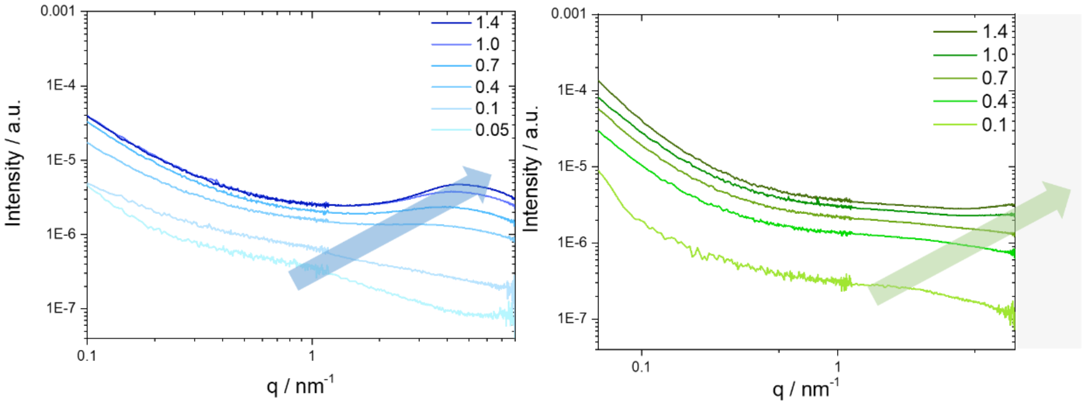

Figure S7. Reproducibility of SAXS measurements in different runs using the example of $FASnI_3$ in DMSO (1 M). Beam damage can be excluded.

Figure S8. SAXS scattering curves for $FASnI_3$ in different concentrations in DMF (left) vs DMSO (right). Evolution of the structure factor peak goes beyond the limited window of the respected q-range in SAXS. Still, the trend is identifiable.

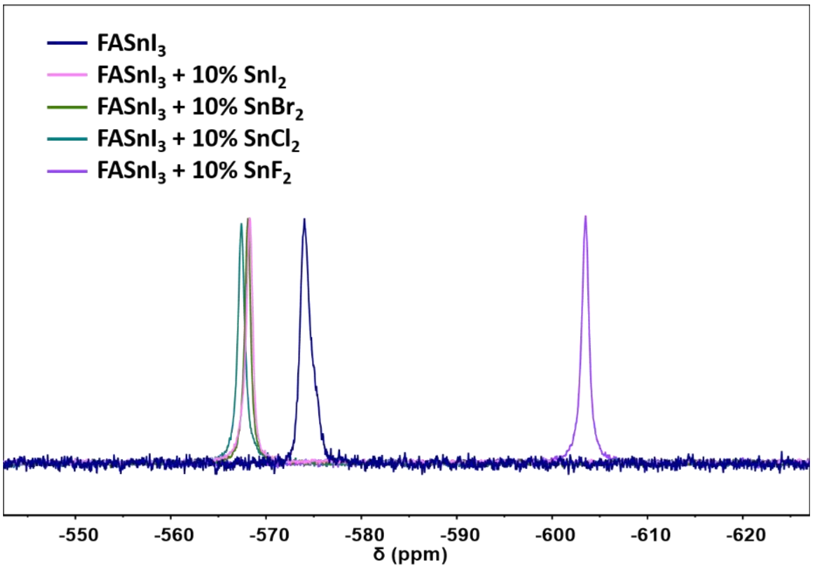

Figure S9. SAXS scattering curves of $FASnI_3$ solution in DMSO compared to $FASnI_3$ with 10 % of SnF_2, $SnCl_2$, $SnBr_2$ and SnI_2. In the small inset, the $FASnI_3$ scattering curve is plotted in direct comparison to an aged solution.

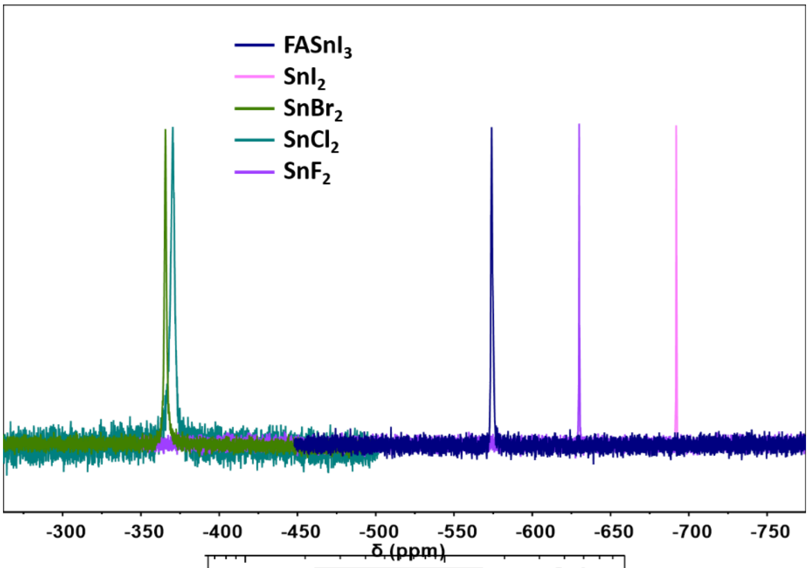

Figure S10. ^{119}Sn-NMR of 1 M $FASnI_3$ solutions with 10 % molar concentration of SnX_2 additives (X = F, Cl, Br, I).

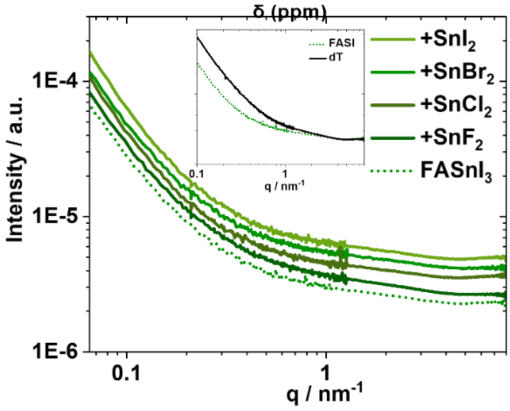

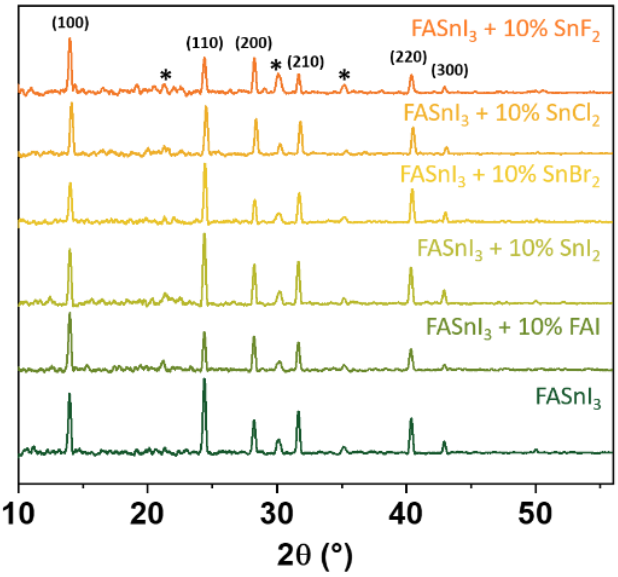

Figure S11. ^{119}Sn-NMR of $FASnI_3$, SnF_2, $SnCl_2$, $SnBr_2$ and SnI_2 in DMSO. $FASnI_3$ and SnI_2 were prepared at 1 M, while SnF_2, $SnCl_2$ and $SnBr_2$ were at 0.5 M.

Figure S12. From left to right, heated 1 M $FASnI_3$ solutions in DMSO first saturated with CsF and NaF, then heated and first heated, then saturated with NaF and CsF.

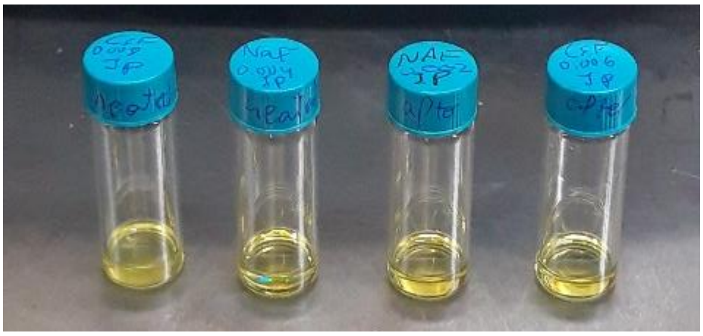

Figure S13. XRD of thin films of pristine $FASnI_3$ and containing a 10 % excess of FAI, SnI_2, $SnBr_2$, $SnCl_2$ and SnF_2.

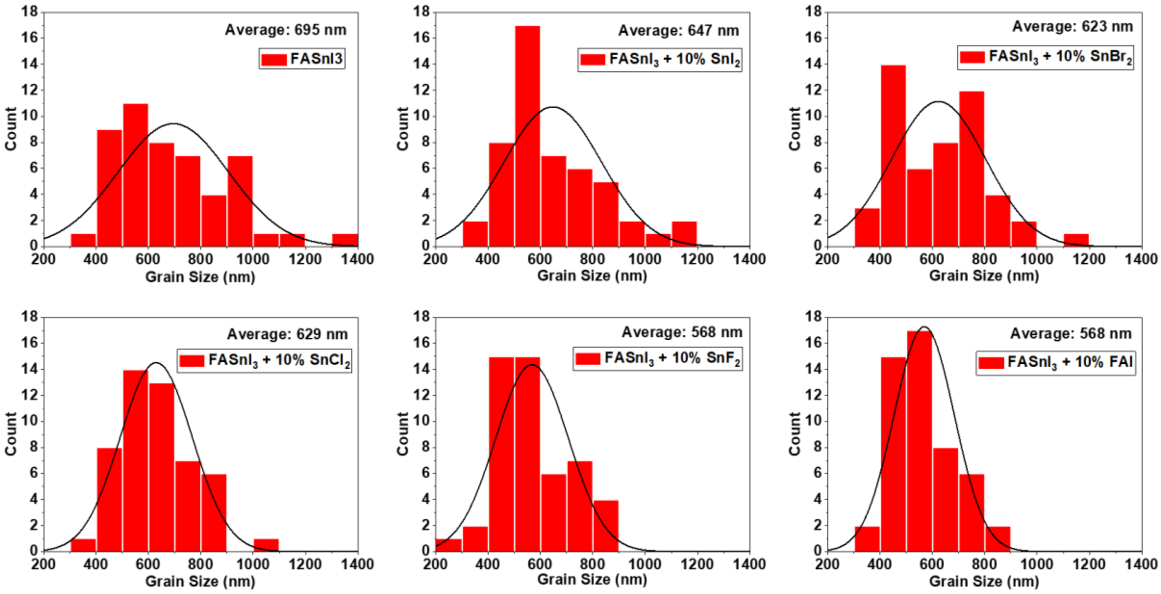

Figure S14. SEM of thin films of pristine $FASnI_3$ and containing a 10 % excess of FAI, SnI_2, $SnBr_2$, $SnCl_2$ and SnF_2.

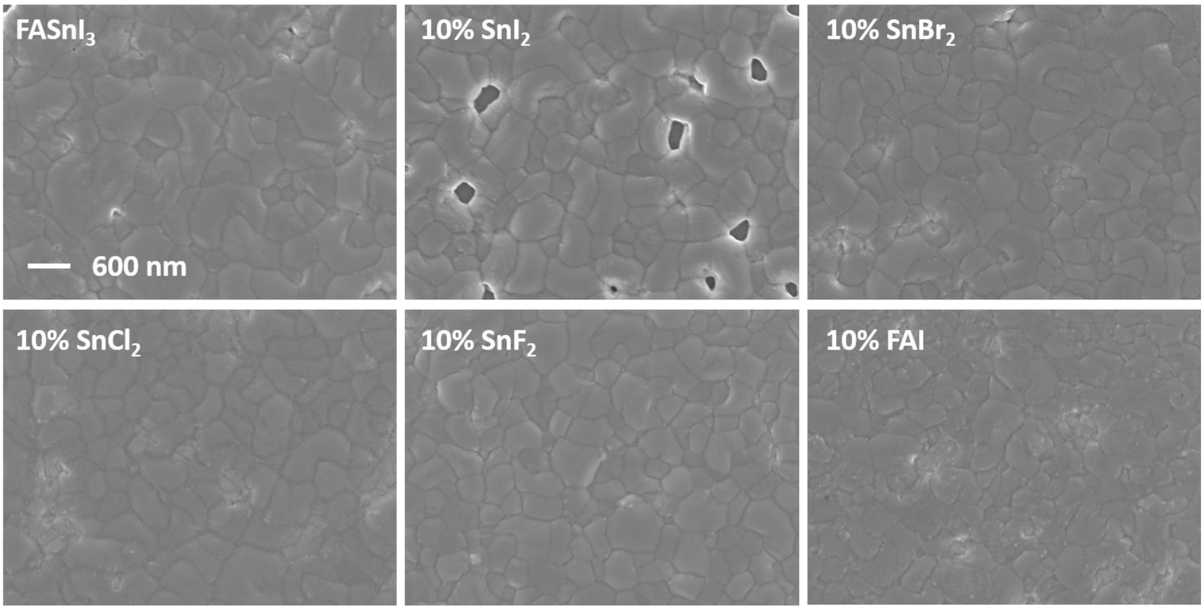

Figure S15. Grain size distribution from the SEM of thin films of pristine $FASnI_3$ and containing a 10 % excess of FAI, SnI_2, $SnBr_2$, $SnCl_2$ and SnF_2. 50 grains were measured for the statistics of each sample.

Figure S16.

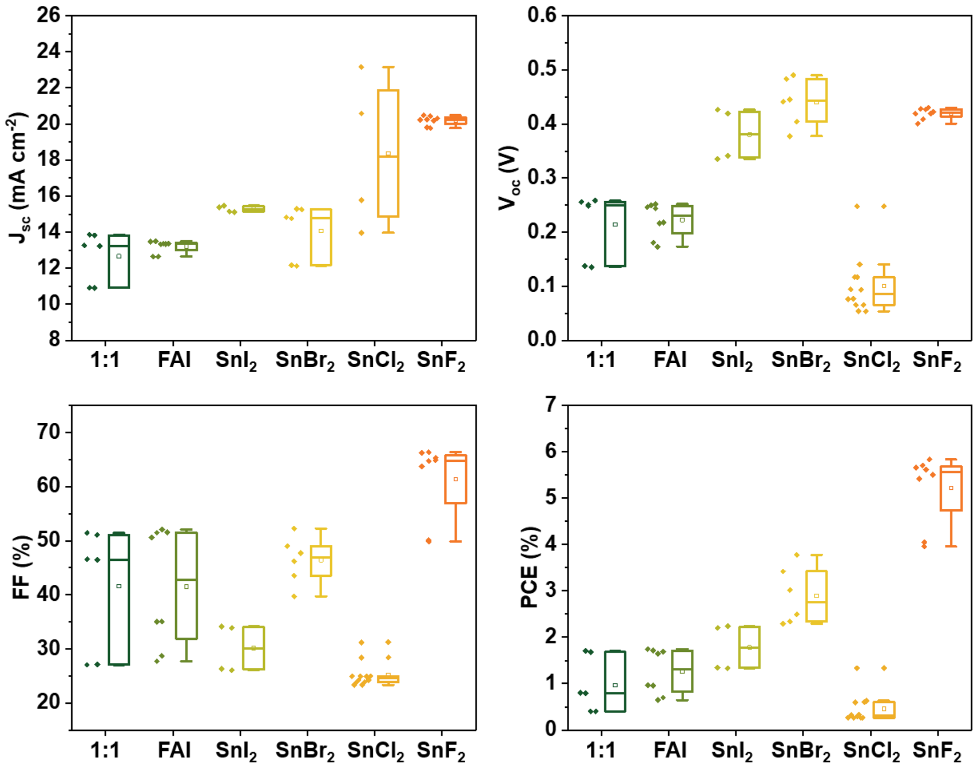

Performance of solar cells based on pristine $FASnI_3$ and containing a 10 % excess of FAI, SnI_2, $SnBr_2$, $SnCl_2$ and SnF_2.

References

[1] M. Krumrey, G. Ulm, *Nucl. Instruments Methods Phys. Res. Sect. A Accel. Spectrometers, Detect. Assoc. Equip.* 2001, *467*, 1175–1178.
[2] A. Hoell, I. Zizak, H. Bieder, L. Mokrani, DE102006029449.
[3] U. Keiderling, *Appl. Phys. A* **2002**, *74*, 1455–1457.
[4] I. Breßler, J. Kohlbrecher, A. F. Thünemann, *J. Appl. Crystallogr.* **2015**, *48*, 1587–1598.
[5] A. Vrij, J. W. Jansen, J. K. G. Dhont, C. Pathmamanoharan, M. M. Kops-Werkhoven, H. M. Fijnaut, *Faraday Discuss. Chem. Soc.* **1983**, *76*, 19–35.
[6] D. Orsi, A. Fluerasu, A. Moussaïd, F. Zontone, L. Cristofolini, A. Madsen, *Phys. Rev. E* **2012**, *85*, 1–9.
[7] D. Frenkel, S. Auer, *Nature* **2001**, *409*, 1020–1023.
[8] J .K. Percus, G. Yevick, *J. Phys. Rev.* **1958**, *110*, 1–13.
[9] A. Vrij, *J. Chem. Phys.* **1979**, *71*, 3267–3270.
[10] M. Gorgoi, S. Svensson, F. Schäfers, G. Öhrwall, M. Mertin, P. Bressler, O. Karis, H. Siegbahn, A. Sandell, H. Rensmo, W. Doherty, C. Jung, W. Braun, W. Eberhardt, *Nucl. Instrum. Methods Phys. Res., Sect. A* **2009**, *601*, 48.
[11] F. Schaefers, M. Mertin, M. Gorgoi, *Rev. Sci. Instrum.* **2007**, *78*, 123102.
[12] S. Tougaard, QUASES-IMFP-TPP2M program. Quases-Tougaard Inc 2002.
[13] S. Tanuma, C. J. Powell, D. R. Penn, *Surf. Interface Anal.* **1994**, *21*, 165.
[14] J. Pascual, G. Nasti, M. H. Aldamasy, J. A. Smith, M. Flatken, N. Phung, D. Di Girolamo, S.-H. Turren-Cruz, M. Li, A. Dallmann, R. Avolio, A. Abate, *Mater. Adv.* **2020**, *1*, 1066–1070.

Author Contributions

J.P., M.F.: Conceptualization, Investigation, Data Curation, Writing - Original Draft, Visualization; R.F.: Investigation, Data Curation, Writing - Original Draft; G.L., S.-H.T.-C., M.H.A., C.H., M.L., D.D.G., G.N., A.D., A.H.: Investigation, Writing - Review & Editing; R.G.W., M.B.: Data Curation, Writing - Review & Editing; A.A.: Supervision, Resources, Writing - Review & Editing. The manuscript was written through contributions of all authors. All authors have approved the final version of the manuscript. ‡These authors contributed equally.
